# Muscle architecture, growth, and biological Remodelling in cerebral palsy: a narrative review

**DOI:** 10.1186/s12891-022-05110-5

**Published:** 2022-03-10

**Authors:** Geoffrey G. Handsfield, Sîan Williams, Stephanie Khuu, Glen Lichtwark, N. Susan Stott

**Affiliations:** 1 Auckland Bioengineering Institute, University of Auckland, Auckland CBD, Auckland, 1010 New Zealand; 2 Liggins Institute, University of Auckland, Auckland CBD, Auckland, 1010 New Zealand; 3 School of Allied Health, Curtin University, Kent St, Bentley, WA 6102 Australia; 4 School of Human Movement and Nutrition Sciences, University of Queensland, QLD, St Lucia, 4072 Australia; 5 Department of Surgery, Faculty of Medical and Health Sciences, University of Auckland, Auckland CBD, Auckland, 1010 New Zealand

## Abstract

Cerebral palsy (CP) is caused by a static lesion to the brain occurring in utero or up to the first 2 years of life; it often manifests as musculoskeletal impairments and movement disorders including spasticity and contractures. Variable manifestation of the pathology across individuals, coupled with differing mechanics and treatments, leads to a heterogeneous collection of clinical phenotypes that affect muscles and individuals differently. Growth of muscles in CP deviates from typical development, evident as early as 15 months of age. Muscles in CP may be reduced in volume by as much as 40%, may be shorter in length, present longer tendons, and may have fewer sarcomeres in series that are overstretched compared to typical. Macroscale and functional deficits are likely mediated by dysfunction at the cellular level, which manifests as impaired growth. Within muscle fibres, satellite cells are decreased by as much as 40–70% and the regenerative capacity of remaining satellite cells appears compromised. Impaired muscle regeneration in CP is coupled with extracellular matrix expansion and increased pro-inflammatory gene expression; resultant muscles are smaller, stiffer, and weaker than typical muscle. These differences may contribute to individuals with CP participating in less physical activity, thus decreasing opportunities for mechanical loading, commencing a vicious cycle of muscle disuse and secondary sarcopenia. This narrative review describes the effects of CP on skeletal muscles encompassing substantive changes from whole muscle function to cell-level effects and the effects of common treatments. We discuss growth and mechanics of skeletal muscles in CP and propose areas where future work is needed to understand these interactions, particularly the link between neural insult and cell-level manifestation of CP.

## Background

### Cerebral palsy

Cerebral palsy (CP) is one of the most common causes of acquired physical disability in childhood, and the most common cause of physical disability in developed nations [1], with an occurrence of between 1.5 to 4 per 1000 live births [2–5]. Worldwide, there are estimated to be 17 million people living with CP, at least 80% of whom will live into their sixth decade [6]. Cerebral palsy was first identified as a separate disorder in the 1800s, with seminal works by orthopaedic surgeon Dr. William Little describing the secondary musculoskeletal deformities consequent to abnormal events at birth [7, 8]. Early definitions attempted to link cerebral palsy with specific pathologies and etiologies [9]. However, a more encompassing definition came in 1964, when Bax described cerebral palsy as ‘a disorder of posture and movement due to a defect or lesion of the immature brain’ [10]. Mutch et al. in the 1990s extended this definition of CP to ‘an umbrella term, covering a group of non-progressive, but often changing, motor impairment syndromes secondary to lesions or anomalies of the brain arising in the early stages of development’ [11]. This definition recognised that CP covers many etiologies and that, while the disorder of posture and movement is permanent and unchanging, the motor impairments are often progressive. In 2007, a group of international collaborators refined this further to recognise other disturbances often seen in CP [12]. Here, CP was defined as “a group of permanent disorders of the development of movement and posture, causing activity limitations that are attributed to non-progressive disturbances that occurred in the developing foetal or infant brain.” Rosenbaum et al. added, “The motor disorders of CP are often accompanied by disturbances of sensation, perception, cognition, communication, behaviour, by epilepsy and by secondary musculoskeletal problems.” [12]. While this remains a widely used definition, challenges remain around the heterogeneity of presentation, the interpretation of the term ‘non-progressive disturbances’ and the age at which cerebral palsy can be acquired. While the etiologies of CP are many, abnormal neuroimaging findings are seen in between 80 and 90% of children with CP [13–15], with changes in neuroimaging broadly linked to different presentations. There is not a singular diagnostic test for CP; however, a combination of early cranial Magnetic Resonance Imaging (MRI), together with the Hammersmith Infant Neurological Examination and General Movements assessment, has a 97% sensitivity to detection of high CP risk before the age of 6 months [16].

Individuals with CP have variable motor impairments, topographical involvement, and levels of functional ability. Classification of the motor impairment has historically been based on the description of the movement and posture disorder and the limbs affected. Two main movement disorders are described: pyramidal (spastic CP) and extrapyramidal (ataxic, athetoid or dystonic CP). Spasticity is defined inconsistently in the literature, but based on the SPASM Consortium definition, broadly includes “disordered sensori-motor control, resulting from an upper motor neuron lesion, presenting as intermittent or sustained involuntary activation of muscles” [17], or “all intermittent or sustained involuntary hyperactivity of a skeletal muscle associated with lesions of the descending motor pathways” [18]. Bar-On et al. [19] have offered that a quantitative and specific definition of spasticity is important, suggesting a return to the classical Lance definition [20] of “a velocity dependent increase in stretch reflex” with renewed emphasis on techniques for specifically measuring this aspect of hypertonicity. Contribution to this debate is outside of the scope of the present article, but we note that there is increasing convergence on a common definition by both researchers and clinical practitioners.

Classification in CP uses different descriptors and may assess different aspects of the clinical manifestation such as topography of affected limbs, severity of movement impairment, or upper limb manual ability. For instance, regarding topographical classification, unilateral involvement of an arm and leg is termed hemiplegia, predominant lower limb involvement is termed diplegia, and involvement of all limbs is termed quadriplegia [5, 21]. While historically widely used, this topographical classification system has low reliability and validity across observers [22] and arguments have been made for its phasing out due to imprecision of description [23]. The Surveillance of Cerebral Palsy in Europe (SCPE) classification divides the movement impairment in CP into spastic, dyskinetic and ataxic forms, with involvement of either one (unilateral) or both (bilateral) sides, and has been shown to have higher reliability when standardised data collection forms are used to guide the reviewer [24, 25]. The Gross Motor Function Classification System (GMFCS) [26] is graded from levels I to V and describes usual gross motor function. The GMFCS uses descriptors at each level defining motor abilities applicable to each level. Children who function at GMFCS level I are independently ambulatory across all surfaces with difficulties only with coordination and balance. Conversely, children who function at GMFCS level V lack head control and require support with all tasks of daily living. Overall, approximately 60% of children with CP are independently ambulatory (GMFCS levels I to II), 11% use some form of walking aides (GMFCS level III), and 29% are predominantly wheelchair users (GMFCS levels IV and V) [27]. Similarly, other common functional classification systems such as the the Manual Ability Classification System (MACS), the Communication Function Classification System (CFCS), and the Eating and Drinking Ability Classification System (EDACS) also provide reliable classifcations (also scaled by levels I-V) of the functional abilities of individuals with CP [28].

### Motor development and musculoskeletal growth in typically developing infants and infants with cerebral palsy

Limb movements are largely involuntary before three months of age and occur with other movements of the body. These ‘general movements’ – movements in which all of the body participates – gradually give way to goal-directed arm and leg movements by 3 to 5 months of age, with control being attained centrally (e.g. arms) before peripherally (e.g. hands) [29]. Conversely in the infant with CP, achievement of motor milestones is delayed and general movements that are reduced in complexity and variation are seen in the first few months of life [30]. Typically developing (TD) muscle growth has not been mapped over time in infants but, by 15 months of age, children with CP have medial gastrocnemius muscles that are already smaller than those of their peers, a finding more evident in those children who are least mobile [31]. This reduction in growth, due to smaller width of muscle fibres, suggests that early typical levels of neural activity and physical activity are critical for normal infant muscle growth. Consistent with this hypothesis, animal models of brachial plexus palsy have shown that division of upper plexus in neonatal rats rapidly leads to reduced cross-sectional and longitudinal growth of C5/C6 innervated muscles with increased fibrosis, fat infiltration, and shoulder or elbow contracture development [32]. While a specific animal model of CP that recapitulates the motor phenotype remains elusive, it is likely that these results from brachial plexus palsy animal models will also apply to CP.

**Fig. 1:**
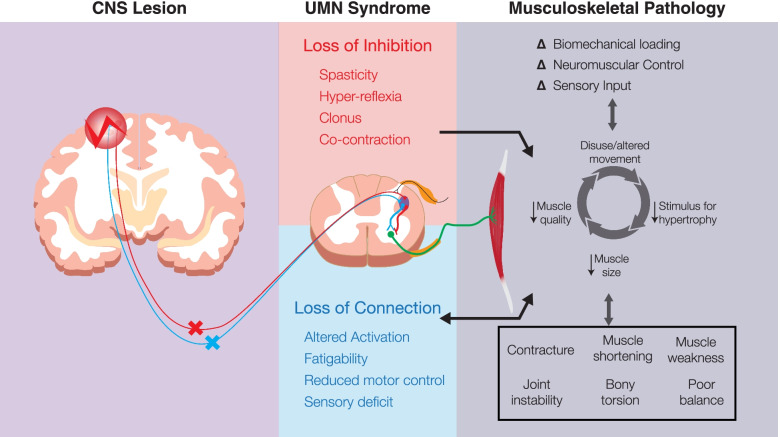
Cerebral Palsy is caused by a lesion in the developing brain, which causes downstream neuromuscular effects. Musculoskeletal effects of CP worsen progressively over time, even though the neural lesion is static. The musculoskeletal pathology may progress in a vicious cycle, where e.g. diminished loading promotes muscle weakness, which further promotes diminished loading. Here we represent both loss of inhibition and loss of connection, and propose that debate and discussion is warranted on the influence that each of these has on the clinical manifestation of CP. Figure our own, created using Adobe Illustrator (Adobe, Inc., San Jose, CA, USA) under the University of Auckland site license

As the child with CP ages, further reduction in muscle growth in involved limbs leads to muscles in adulthood that are smaller, shorter, and weaker, with reduced exercise tolerance and greater fatigue [33–37]. These features are additive to the neurologic impairments in CP, which include hypertonia, hyperkinesis and altered motor control [38, 39] (Fig. 1). Joint and muscle range of motion (ROM) can become increasingly limited over time, and secondary muscle and joint contractures develop, resulting in pain, hip subluxations, and scoliosis [38, 40]. Altered musculoskeletal function becomes evident early in life and typically worsens over time, greatly impacting the lives of individuals with CP across their lifespan [41]. The role of musculoskeletal growth in CP is under-studied; the effects of muscles and bones that are growing, in conjunction with neural impairments and altered mechanics, may explain some of the progression of symptoms in CP. Beyond this, the link between the neural insult and the cellular myopathies of cerebral palsy is unclear, but there may be a central mechanism describing how cellular muscular components are altered by the neural lesion in CP. These questions are ripe for further work. Broadly, optimisation of muscle health in CP requires an understanding of the complexities of muscle morphology and dynamics, and warrant an elucidation of the effects of the pathology on the musculoskeletal system, which in turn play a significant role in the manifestation of CP.

This focused narrative review provides an overview of recent literature on the effects of cerebral palsy on muscle growth and development and how this influences the progression of cerebral palsy and impacts the lives of those with this condition. We present current descriptions of the musculoskeletal aspects of cerebral palsy, focusing primarily on skeletal muscle, and assessing size scales from the whole patient down to the cellular level. We focus attention on the role of musculoskeletal growth, and how this may interact with other aspects of CP to progress symptoms over time.

### Effects of cerebral palsy on muscle size and architecture

Muscle size and architecture are measurable features at the macro- and meso-scale that govern a muscle’s functional capacity [42]. Deficits to muscle size and architecture indicate functional loss. As a result, previous work has used medical imaging to investigate differences in size and architecture between typical and CP groups [41, 43–51]. Common findings include deficits in muscle volume and cross-sectional area [41, 43–50], and overstretched sarcomeres [42, 52, 53]. Here, we review this literature and propose that these changes to muscle may result from dysfunctional growth at the cellular level which limits potential increases in physiological cross-sectional area that may otherwise occur in response to aging and mechanical stimulation of exercise on the muscle; dysfunctional cellular growth may also limit serial sarcomerogenesis for reasons that, at present, remain hypothetical.

#### Muscle size

Muscle volume represents gross size and can be determined from medical imaging. Geometrically, muscle volume is the three-dimensional representation of muscle length and cross-sectional area, or thickness in sagittal and coronal planes. Given that all three measures may be reduced in muscles from individuals with CP, it is unsurprising that muscle volume is markedly reduced compared to TD muscles [41, 43–49]. Percent differences in volumes vary widely across individuals and muscles but may be as much as 43% reduced [47, 49]. While individuals with CP are often smaller in height and mass than TD individuals, small volumes are not explained by the smaller statures of individuals with CP [49]. Current data suggests that the greatest reductions are associated with reduced overall function according to GMFCS levels [54, 55].

**Fig. 2:**
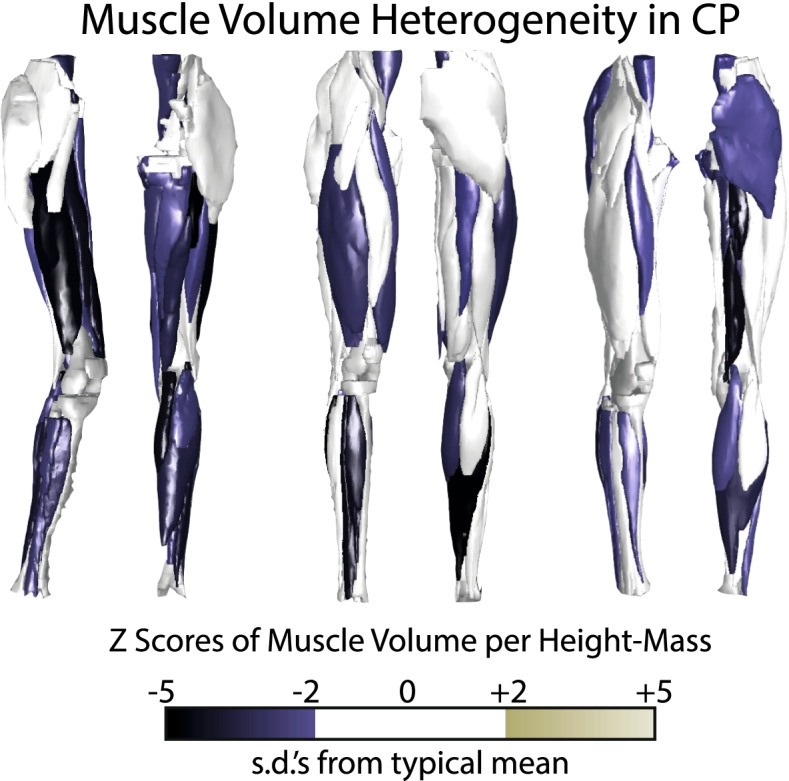
Z-scores of height-mass normalized muscle volumes reveal the heterogeneity of muscle size deficits in CP. Volume deficits are non-uniform within limbs, and muscle deficit profiles are non-uniform across individuals with CP, emphasizing the importance of evaluating the pathology on a patient-specific and muscle specific basis. Figure redrawn with permission from Handsfield et al .[49], using Adobe Illustrator (Adobe, Inc., San Jose, CA, USA) under the University of Auckland site license

Another aspect of muscle size in CP is the heterogeneity of expression of volume deficits (Fig. 2) [49, 56]. In a study of 35 lower limb muscles in 18 adolescents, Handsfield et al. demonstrated that muscles were not uniformly reduced in volume in individuals with CP, implying that not all muscles are affected equally by the neurologic condition [49]. The profile of muscle sizes was also significantly different between individuals with CP, implying a unique manifestation for each individual. In light of this, it should not be surprising that there are between-study differences in volume or length deficits reported, e.g. soleus volumes reported in Oberhofer et al. [45] vs Handsfield et al. [49]. Differences between studies such as these are a feature of CP and reinforce the importance of treating CP subject-specifically [56].

#### Muscle-tendon length, sarcomere length, and Pennation angle

**Fig. 3:**
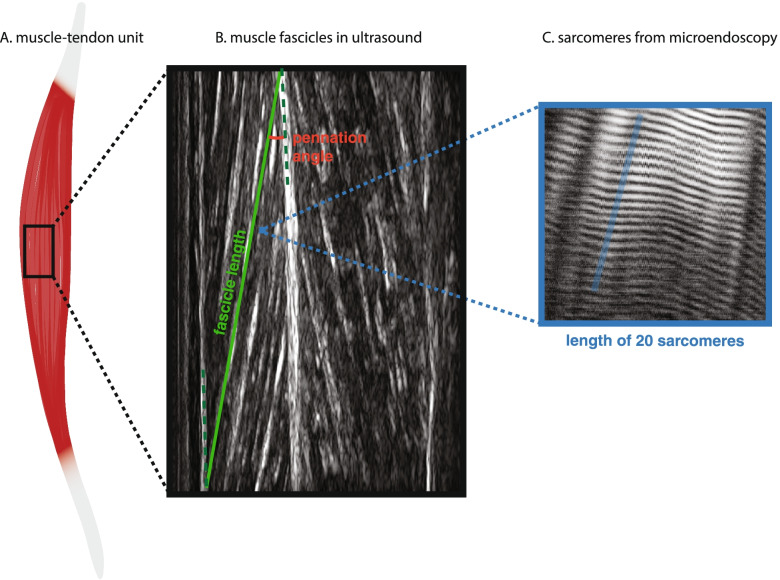
CP affects muscle structure at the level of the muscle-tendon unit (**A**), the fascicles (**B**), and sarcomeres (**C**). **A**: Muscle belly lengths have been observed as shorter in CP, while tendons have been reported as longer. **B**: Fascicle lengths (green line) are reported to be shorter or similar in length between individuals with CP and TD cohorts, while pennation angles (red) are reported as similar or smaller in individuals with CP, sometimes with joint angle dependencies. **C**: microendoscopy image shows a muscle fibre and sarcomere striation pattern, blue line captures ~ 20 sarcomeres in series, reported to be longer in individuals with CP. Figure our own, created using Adobe Illustrator (Adobe, Inc., San Jose, CA, USA) under the University of Auckland site license

The muscle-tendon unit (MTU) (Fig. 3) comprises the muscle belly and the tendons. Compared to typical muscles, affected muscle bellies in CP are generally shorter, smaller in thickness and cross-section, and have longer tendons [41, 49, 50]. Overall, the MTU of affected muscles are likely shorter in CP muscles at the point of passive-force generation, although this is muscle- and patient-specific, and varies with age and severity of CP.

Ultrasound studies on plantarflexor muscles have shown similarity between typical cohorts and cohorts with CP for both muscle belly length and fascicle length^1^ (Fig. 3) [44, 57–59] or alternatively shorter in muscles from individuals with CP [60]. Using diffusion tensor imaging, Sahrmann et al. [50] showed no significant differences in soleus muscle fascicle lengths between a group of typical adolescents and a group with CP; D’Souza et al. [51] showed significantly shorter fascicles in medial gastrocnemius muscles from individuals with CP compared to typical. Across imaging modalities, fascicle length differences range from no difference [46, 50, 61] to up to 25% shorter in muscles from individuals with CP [60, 62] or in some cases beyond 40% [63].

At the micro-scale, the fundamental unit of muscle is the sarcomere (Fig. 3). Human muscle fibres are composed of tens of thousands of sarcomeres in series [64, 65]. A muscle’s functional ROM is affected by the number of sarcomeres in series, with fewer sarcomeres contributing to a smaller overall muscle excursion. It is difficult to know the resting length or number of sarcomeres in individual muscles without direct investigation. Intraoperative methods to determine sarcomere lengths [42, 66] have reported generally consistent findings that sarcomeres from muscles of individuals with CP are significantly longer and fewer in number than in TD muscle. However, previous studies either lacked a consistent control group [52, 67] or measurements were made at a prescribed joint angle where passive tension may have been different between groups [42, 53]. Furthermore, indirect measures of the forces generated by muscle at different lengths [68–71], have suggested similarity in sarcomere force-length relationships between groups with CP and TD groups. Despite these discrepancies, direct evidence seems to suggest that CP is associated with fewer sarcomeres in series and longer sarcomere lengths relative to fascicle length (Fig. 3), which conceivably plays some role in muscle contracture. Novel methods to assess sarcomere length in vivo, for example microendoscopy [64, 72, 73], used in conjunction with macro-level imaging, such as ultrasound or diffusion tensor MRI, may provide greater understanding of muscle fibre level adaptations in CP.

Tendon, aponeurosis and other connective tissue also play a role in the generation of passive tension in the MTUs. Tendons in the lower limb of individuals with CP have been observed to be on the order of 10% longer than TD controls [71, 74, 75], which may be a compensation to shortened muscle bellies. However, increased tendon lengths appear insufficient to fully compensate for the shorter fascicle lengths, thus leaving stretched sarcomeres and a muscle which is overall tighter and less extensible than typical [76–78]. In other populations where atrophy is known to occur, e.g. ageing muscle, tendinous tissues are presumed to adapt to allow a normal ROM [79]. There is currently little understanding around how tendinous tissue adapts in individuals with CP and whether this allows more optimal force generation at shorter overall MTU lengths.

A muscle’s pennation angle is defined as the angle between the muscle fibre direction and the external tendon direction (see Fig. 3). Studies using ultrasound and diffusion tensor MRI have explored pennation angles in muscles from individuals with hemiplegic and diplegic CP and from typical controls. Results have been mixed with reports of increases [60, 80, 81], decreases [57], and no significant differences [50, 51, 62, 74, 82]. It should not be surprising that results vary on this question given the general heterogeneity of CP pathology across both muscles and individuals [49]. Diminished pennation angles have been associated with sarcopenia and atrophy [83–85], although in individuals with CP this may be complicated by altered sarcomere numbers and tendon lengths. Some of the aforementioned studies found a dependency on joint position [57, 76], suggesting a difference in resting joint angle that is consistent with an overall tighter MTU in CP.

#### Muscle physiological cross sectional area

**Fig. 4:**
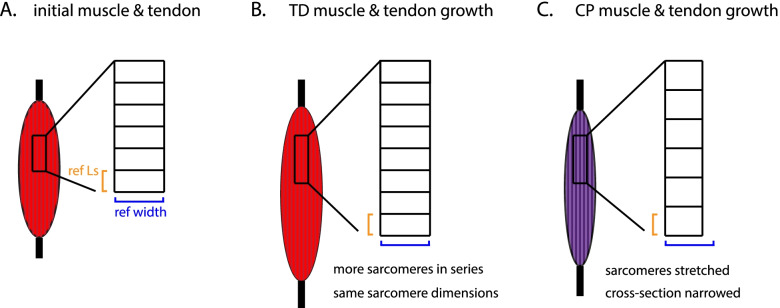
Hypothetical mechanism for increased sarcomere length and reduced apparent PCSA in CP muscle (inset represents sarcomere structures within muscles). A typical muscle grows in length by adding sarcomeres in series (**A** and **B**). Sarcomere length at a neutral joint angle (Ls) remains constant but the number of sarcomeres in series increases. In individuals with CP, muscles and tendons also lengthen with growth, but sarcomeres stretch out, rather than adding in series, while tendons lengthen (**A** and **C**). This effect may contribute to reduced cross-sections (see ref. width in **C**); a muscle with 20% longer sarcomeres is 20% narrower in cross-section because of the isovolumetric property of muscle. Figure our own, created using Adobe Illustrator (Adobe, Inc., San Jose, CA, USA) under the University of Auckland site license

Physiological cross sectional area (PCSA) is the cross-sectional area perpendicular to muscle fibre direction when the muscle is at optimal sarcomere length. For typical individuals, PCSA is a proxy for maximum isometric force [86]. Because PCSA is tightly linked to force production, it is an important measurement for understanding strength deficits in CP, bearing in mind that muscle quality, i.e. specific tension, may also be impaired in individuals with CP [55, 87–90]. Previous authors have used muscle thickness and anatomical cross-sectional areas as indicators of PCSA, largely reporting deficits among individuals with CP [31, 62]. Two recent studies using diffusion tensor MRI showed reductions in cross-sections of around 40% in the soleus [50] and 10% in the gastrocnemius [51] in individuals with CP compared to TD. Neither of these groups determined sarcomere lengths as part of their study. Because muscles are isovolumetric when they stretch and contract, CP muscles with 20% longer sarcomeres would also be 20% smaller in cross-section at that position. Thus, some of the reduction in observed PCSA could be accounted for by overly long sarcomeres in CP (Fig. 4). Studies involving concomitant fascicle length assessment and sarcomere length determination are an important future work to resolve this question. It is possible however that sarcomere length increases do not fully account for observed reductions in muscle cross-sections, indicating structural muscle weakness as a feature of CP. Continued work is warranted to connect sarcomere lengths, fascicle lengths at neutral joint positions, and PCSA in CP and TD populations.

In summary, CP is associated with muscles that may or may not be shorter in fascicle length compared to typical; the limited evidence on sarcomere lengths suggests that there are fewer sarcomeres in series that are stretched out in muscles from individuals with CP (Fig. 4); and reduced thicknesses and cross-sectional areas indicate an impaired strength capacity among individuals with CP. These changes may be accompanied by longer tendons. What remains unclear is whether smaller muscles are the result of a process of atrophy or a lack of growth. Atrophy implies that the muscles are becoming smaller over time, while lack of growth suggests that the muscles are failing to develop adequately alongside a growing body. One mechanism linking these observations concerns the lengthening of bones in growing children and adolescents. The process of bone lengthening by growth will mechanically lengthen the muscles and tendons. In TD children, muscles respond by adding sarcomeres in series and growing in length (Fig. 4). If CP is associated with a failure of muscles to add sarcomeres in series, the muscles accommodate the growing skeleton via overstretched sarcomeres and tendons (Fig. 4). An alternative explanation is that fascicle excursion is restricted by a stiffer extracellular matrix [53] and restricted motion then inhibits longitudinal growth of the muscle. There is strong evidence that muscle growth is impaired in CP for both longitudinal growth (addition of sarcomeres in series) [51, 53, 91] and cross-sectional growth (increases in PCSA) [49–51]. Other musculoskeletal effects of CP, e.g. longer tendons, may be explained as consequences of these more primary causes. We propose that the impaired capacity for growth in CP manifests at the cellular level, a hypothesis that will be discussed in detail in the next section. The link between the neural lesion and the altered cellular environment remains elusive, but is an important area for further directed study.

### Muscle cellular physiology and growth

**Fig. 5:**
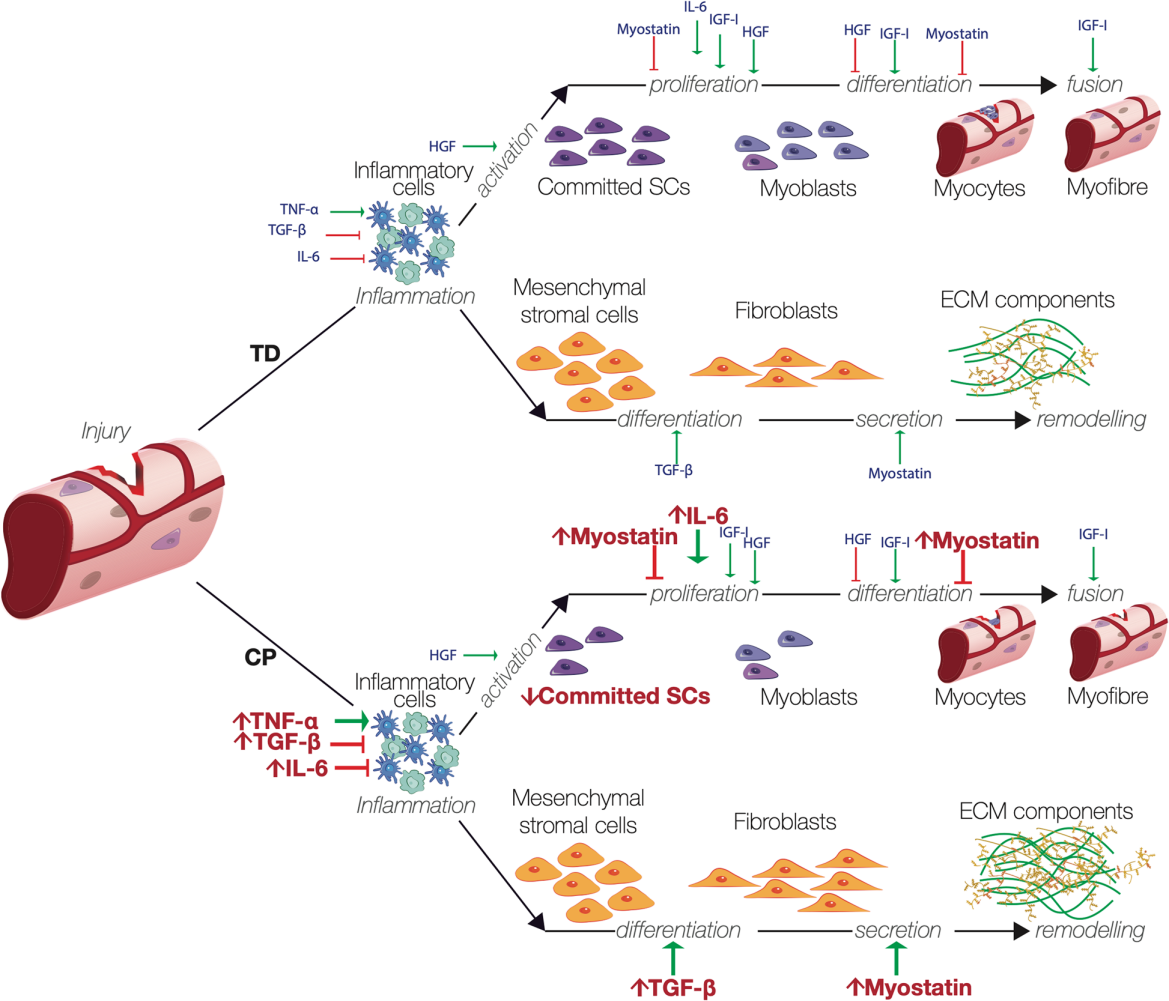
Mechanical injury is the first step in muscle regeneration and growth and initiates a cascade of cellular and molecular events in skeletal muscle. Inflammatory cells debride tissue and clear damaged muscle cells. Release of appropriate cytokines activates resident satellite cells to repair the fibre while fibroblasts remodel the ECM. CP muscle displays increased expression of TNF-a, TGF-B, IL-6 and myostatin, and fewer SCs. Increases in fibroblast activity result in smaller fibres and excess ECM. Figure by Stephanie Khuu/ ﻿CC BY 4.0

The cellular environment and morphology of skeletal muscle is altered in CP compared to typical [92]. Common histological observations of muscles from individuals with CP include altered fibre type distribution, increased variability in fibre size, rounding of muscle fibre cross-sections [93, 94], increased lipid composition [94–96], and excess connective tissue [53, 95, 96]. These pathological changes are linked to reduced regenerative potential and have been correlated with changes in pro-inflammatory cytokines, satellite cell concentration, and fibroblast activity, all of which are important for achieving muscle homeostasis following mechanical damage to muscle [95, 97–99]. The process of muscle regeneration and growth is the subject of ongoing research, but can be summarised by the inter-dependent processes of (1) mechanical injury; (2) inflammation marked by the clearance of necrotic tissue by myeloid cells; (3) repair marked by satellite cell activation and differentiation into myoblasts and myocytes, which fuse to repair or generate muscle cells; and (4) remodelling of the extracellular matrix (ECM) by fibroblasts (Fig. 5).

Aberrant changes in levels of cytokines have far-reaching effects on the whole muscle as a result of altered regeneration. Elevated levels of pro-inflammatory cytokines interleukin-6 (IL-6), tumor necrosis factor alpha (TNF-α) have been found in muscles from individuals with CP and are linked to reductions in protein synthesis, stunted growth, and muscle atrophy [95]. Transforming growth factor beta (TGF-ß) is elevated in CP muscles compared to typical, as is myostatin, a negative regulator of skeletal muscle mass and promoter of protein degradation [95, 100]. Myostatin is up-regulated by increased expression of TGF-ß, and impairs satellite cell proliferation during growth and repair of skeletal muscle by signalling quiescence of satellite cells [101–104] while simultaneously enhancing fibroblast and ECM protein proliferation [105].

Satellite cells (SCs) are resident stem cells considered to be the primary agents of repair in skeletal muscle. Reductions in SC concentration of 40–70% have been reported for muscles from individuals with CP compared to typical [91, 95]. In addition to a loss of SCs in CP muscle, the regenerative ability of remaining SCs may be compromised. Cultured myoblasts derived from SCs from individuals with CP show down-regulated markers of differentiation and an ~ 85% decrease in the rate of fusion and myotube formation [97]. The decrease in both concentration and efficacy of SCs contributes to a diminished regenerative potential in the muscle environment in CP. However, the mechanistic role of SCs in growth and repair of CP musculature is not well understood. For instance, it may be that SCs are more pertinent to hypertrophy in young muscle for growth and repair, rather than in older individuals; the role of SCs in regulating fibroblast activity during collagen production and ECM remodelling needs further exploration [98, 99].

Healthy muscle regeneration and ECM remodelling are enabled by the reciprocal interactions between SCs and fibroblasts and their related rates of proliferation [106]. ECM is the non-cellular, endomysial connective tissue that surrounds muscle structures [107, 108]. ECM is created and repaired by fibroblasts which secrete ECM components, including collagen [109]. Different ECM compositions have been observed in individuals with and without CP and may account for both loss of function and loss of regenerative potential observed in CP. In connective tissue staining of human flexor carpi ulnaris muscle fibre bundles, tertiary perimyseal collagen was three-fold higher in CP compared to control samples [93]. Moreover, hamstring muscles from children with CP had increased Collagen I and laminin between fibres, indicative of excess connective tissue [53]. Booth et al. found that the level of collagen accumulation in muscle biopsy samples from children with CP was significantly correlated with the Modified Ashworth Scale [110], indicating a link between excessive connective tissue and contractures in CP. On a transcriptional level, CP muscle has increased gene expression for collagen production and pro-inflammatory cytokines compared to TD, both of which can lead to aberrant ECM expansion [95].

Optimal repair of muscle is associated with initial construction of an ECM scaffold, followed by feedback mechanisms to ensure the resolution of fibrosis [111]. When feedback mechanisms are compromised or when injury is repetitive, excess collagen deposition by fibroblasts results in scar tissue formation and loss of contractile function in the area [112]. In cases of spasticity and contractures, it is common to observe increased ECM, fibrosis, and fatty infiltration into muscle [93, 110, 113]. The precise cellular mechanisms for each change remain unknown, but it is thought that increased ECM and fat content may act as a structural barrier to normal growth in CP. Further work is needed on the relationship between muscle components at the cellular level, deposition of structures during muscle and ECM repair following damage, and growth mechanics in CP. An elusive but worthwhile goal for future research is uncovering mechanisms linking the principal neural insult in CP with the commonly observed cellular myopathies—reduced satellite cell number, fibrosis, and reduced muscle fibre area fraction. The elucidation of a neural or neuromuscular antecedent of these myopathies would represent a huge leap forward in understanding and treating spastic CP. In this respect, it is worth considering that CP generally expresses subject-specifically and the field may uncover several unique mechanisms at the neural and cellular scale that individually or collectively contribute to an individual’s specific expression of muscular development.

### Biomechanical loading and growth of muscles in CP across the lifespan

Altered muscle morphology and growth in individuals with CP have biomechanical consequences, which then affect morphology and growth in a cyclical fashion. For example, painful or tiring gait may result in a reduction in voluntary physical activity, which inhibits biomechanical loading to a level that inhibits hypertrophy and results in smaller muscles, further making movement painful or tiring. The cyclical feedback between mechanics, structure, and growth exists at multiple levels and has consequences across the lifespan.

While research is lacking on how morphology and mechanics change over time in individuals with CP compared to typical, piecing together a series of cross-sectional studies and short longitudinal studies suggests muscle growth is impaired from an early age in individuals with CP [114]. Typically within the first years of life, the human body experiences rapid growth in both muscle length and cross-sectional area, enabling attainment and mastery of functional tasks. In individuals with CP, reduced muscle volumes may begin early in childhood. Cross-sectional studies investigating volume of the medial gastrocnemius muscle in infants with CP [31, 115] supports the concept that early growth rate is reduced compared with TD controls [76]. Herskind et al. [31] and Willerslev-Olsen et al. [115] provide evidence that at 12–15 months of age the muscle volume of infants with CP is already reduced compared to TD children. Given that developmental milestones such as standing and walking occur at this age, it may be that the earliest musculoskeletal aspects of CP set the stage for subsequent impaired movements over the lifespan.

**Fig. 6:**
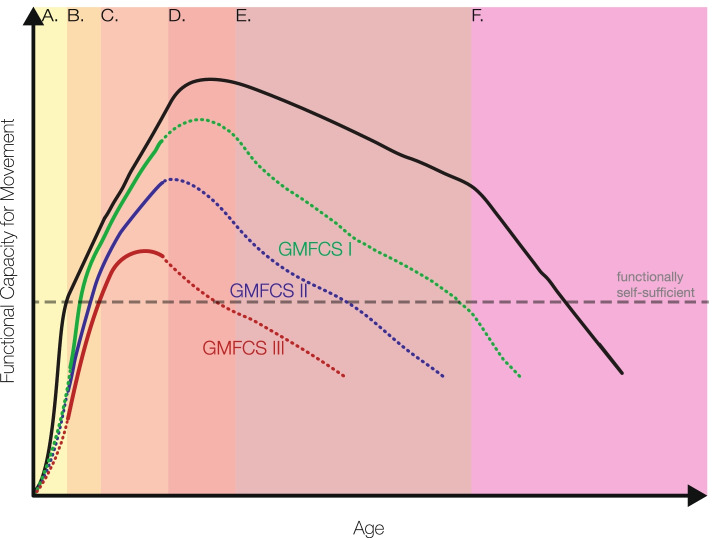
Hypothetical trajectory of muscle function (y axis) across the lifespan (age, x axis) in healthy TD individuals and individuals with CP, plotted for GMFCS level I, II, and III, an expansion of the concept proposed by Shortland [114]. Proposed trajectories transition from solid to dashed lines where literature is sparse. We do not present GMFCS IV-V—a reflection of the dearth of data from these cohorts. The dashed horizontal line represents the hypothetical threshold for functional self-sufficiency. Shaded blocks represent life stages to mark variations in growth rates: **A**) Infancy-early childhood: a period of rapid development, **B**) Childhood: a period of continued improvement, **C**) Adolescence: protracted improvement in TD with lesser gains in CP, **D**) Early-adulthood: reaching our peak, **E**) Adulthood: gradual decline **F**) Older age: rapid decline. Figure our own, created using Adobe Illustrator (Adobe, Inc., San Jose, CA, USA) under the University of Auckland site license

The trajectory of typically developing muscle size and strength steadily increases before reaching a peak in the early to mid-20s [115]. Fitness activities aim to promote development of muscle health during this time and maintain health above the functional strength threshold to combat effects of aging and sarcopenia. Individuals with CP participate in significantly lower rates of physical activity than their TD counterparts [116–121] and entertain fewer opportunities for mechanical loading. This effect increases with decreasing function, according to GMFCS levels [122], potentially contributing to a vicious cycle of ‘disuse’ and secondary sarcopenia, as discussed by Verschuren et al. [37]. Trajectories of muscle volume over time for individuals with CP reaches a lower peak and peaks earlier in life, and is nearer the functional threshold throughout the lifespan (Fig. 6).

Rapid declines in function in otherwise healthy typical individuals is generally expected around the seventh or eighth decade of age [115]. This decline is proposed to occur much earlier in individuals with CP, due to the combined effects of contractures, altered neural drive, reduced mechanical loading, and sub-optimal nutrition [114, 123]. In addition to lower than typical function [124, 125], significant functional declines have also been reported in adolescents and adults with CP [126, 127]. For instance, 31–35% of adults with CP experience a decline in walking ability before the age of 35 [128, 129]. Longitudinal studies support this, finding that walking ability declines with age and are more rapid among individuals with less functional capacity at onset of adulthood [130, 131]. Deterioration in walking function seems to be related to increased pain and fatigue as well as reduced balance [131]. Deficits to neural control, muscle weakness, joint contracture, and spasticity likely interact with the natural progression of ageing to limit functional capacity and physical activity [132]. There is a disappointing lack of research on adults with CP, and even more so in terms of the longitudinal changes of muscle. Interventions and management aimed at improving and maintaining muscle health across the lifespan need to be applicable to all individuals with CP, but perhaps even more so in individuals known to have limited biomechanical loading who are at risk of secondary sarcopenia.

Consideration of biomechanical loading in CP is important in light of early deficits experienced with this pathology and the narrower functional time across the lifespan. Our understanding of cellular physiology and growth in CP is still limited, but coupling this with our understanding of biomechanics at multiple scales may help develop future strategies for maximizing opportunities for muscle growth and development across the lifespan in individuals with CP.

### Impacts to muscles of common treatments for CP

Much of our understanding of muscle in CP is absent knowledge of the natural untreated progression of muscle in CP. Participants with CP involved in research studies have typically been treated with sequential interventions and management approaches, which confound reductionist investigations to fully understand the longterm impacts of the treatments. Here we discuss potential effects of treatment strategies on muscle size and quality.

Pharmacological agents and surgical procedures are frequently used for tone management in CP**,** including Botulinum Neurotoxin A and selective dorsal rhizotomy [133]. Botulinum Neurotoxin A (BoNT-A) injections reduce spasticity with small but variable effects on functional abilities [134, 135]. Recent concerns have emerged that BoNT-A may contribute to muscle atrophy and fibrosis, effects that may outweigh the positive short term reduction in spasticity [136]. Studies investigating echo intensity—a proxy for fatty infiltration—and muscle volume following BoNT-A injection suggests increased fat and decreased muscle volume [55, 88, 137, 138]. However, BoNT-A may not have as profound effects in humans as was previously reported in animal studies [137], and BoNT-A injected muscles are not precluded from also experiencing hypertrophic and functional strength gains following strength-training intervention [139]. Further, BoNT-A does not appear to affect agonist muscles in close proximity to the injected muscle, which may undergo compensatory hypertrophy [138, 139]. However, it bears considering that the chemo-denervation of muscle associated with BoNT-A has been linked to acute atrophy in both animal studies [140, 141], and humans [137, 138, 142, 143]. Beyond this, it should be acknowledged that the long-term effects of BoNT-A remain under-studied, and continued research and some clinical caution are thus appropriate [144, 145].

Selective dorsal rhizotomy (SDR) is a neurosurgical procedure whereby selective sensory nerves running through the spine are irreversibly severed, with the intent to decrease the sensory input of the overactive spastic muscles [146]. The procedure is widely regarded to alleviate muscle tone in the lower limbs of children with spastic diplegia [147–149], and when combined with physiotherapy lead to improvements in gross motor function [147, 150], pain [151], and functional independence [147]. However, researchers highlight the critical importance of careful patient selection to avoid detrimental outcomes of the procedure (i.e. patients with dyskinesia or ataxia and without adequate muscle strength [147, 152]). Other than a reduction in tone, the impact on variables of muscle are under-studied, and evidence on outcomes are still limited to short-term studies.

Muscle-tendon lengthening surgeries are a common muscle surgical intervention for children with CP, with the aim of restoring joint range of motion and normalising gait [153]. Potentially as a result of serial sarcomere loss following surgery, reductions in muscle fibre length and increased deep fascicular-aponeurosis angles have been measured post-surgery [61], as have reductions in muscle belly length [154]. Yet parallel improvements in joint range of motion imply an overall lengthening of the musculotendinous unit, with an increase in tendon length [155]. Though atrophy of the muscle might be expected in the short-term following surgery, middle-term findings are mixed [154, 155], but indicate a recovery and continued increase after such surgery. In contrast, muscle belly length appears to remain reduced relative to bone length; more work is needed in this area to determine the specific impacts of lengthening surgeries on the muscle belly and tendon separately [154, 155].

Treatment strategies for muscle contractures vary in scope and effectiveness. Passive stretching is generally not regarded as useful in contracture prevention or management [156]. Prolonged stretching of MTU components via orthoses or casting may improve joint ROM, but may weaken muscles or adversely affect the muscle belly tendon length ratio [157]. Prolonged use of Ankle-Foot Orthosis may not prevent worsening of a contracture [158]. Studies on bracing have shown increased ROM without increasing muscle belly length via increased tendon extensibility but also reduced muscle volume [159].

Therapeutic interventions targeting muscle weakness and motor control/ neural activation in CP include strength training, mobility training, and functional electrical stimulation. Strength training has successfully induced improvements in muscle volume [139, 160], fascicle length [58], cross-sectional area [58], and muscle thickness [58], while studies employing neuromuscular electrical stimulation have shown improvements in cross-sectional area [161, 162], muscle thickness [162], and muscle volume [163, 164]. It should be noted that gains in muscle architecture and strength may not translate to functional improvements such as walking speed or gross motor function [165, 166]. It is broadly recognized that muscles tend to be weak in cerebral palsy; for this reason, strength training should not be categorically ruled out, but consideration should be put towards the interventions themselves and whether the activities are directed at achieving a desired outcome or functional movement for the patient [167]. In a systematic review of 166 studies, Novak et al. found aerobic fitness training to offer an overall positive effect in CP, in general, but found equivocal results for strength training based on the lack of connection to functional gains [168]; however, in a follow-up review, strength training was found to be a positive intervention in certain contexts (e.g. after casting) and a weak positive to promote fitness, physical activity, and quality of life in general [133]. Novak et al. also reported equivocal results for electrical stimulation [133, 168]. Considering this, we add our support of the principle that targeting muscle weakness may be the most beneficial when there is a focus on therapies that also train motor control and promote a functionally useful movement, such as gait training [166, 169], while also involving mechanical loading.

## Conclusions

Skeletal muscle in CP is associated with observable deficits at several size scales, including reduced muscle volumes, fascicle lengths, and pennation angles, stretched tendons, and fewer sarcomeres in series that are stretched out. Impairments to muscles are heterogeneous in CP and deficits tend to vary across muscles and individuals. Impaired growth in CP is thought to commence at an early age and may relate to deficient concentrations and effectiveness of satellite cells. Higher proportions of ECM and sometimes fatty infiltration are seen in CP, which may be related to altered SC concentrations as well as the dynamic interactions of fibroblasts, SCs, and other cytokines. Further research is needed to understand the nature of the cellular environment, its mechanobiology, and its relationship to the neural insult causing CP, particularly the identification of a mechanism that causes the cellular myopathies as a result of the neural lesion.

CP is complex and cyclical—it is caused by a neural lesion which causes downstream musculoskeletal effects that interact with one another and also interact with the natural processes of growth and aging. While impaired muscular development has been observed at around 15 months of age, atypical movements are noticeable as early as 3 months of age [170], potentially implying that muscle morphology is a downstream effect of altered neural activation and biomechanics, rather than a direct consequence of the neural lesion. Separating these issues in a reductive approach is difficult, but advancements in computational modelling and bioengineering approaches [171, 172] create new advanced possibilities for simulating and understanding these mechanisms.

The very nature of CP makes it subject-specific in its manifestation. Understanding the muscular effects of CP requires approaching the philosophy of CP as an ‘umbrella’ diagnosis or a grouping of pathologies related only in their initial cause and general scope of resultant symptoms. There should be no expectation of uniformity in the expression of muscular pathology. Nevertheless, continued probing of the mechanisms and interactions of the various muscle pathologies associated with CP will enable us to develop new treatments and therapies that are scientifically based and tailored to the individual and their specific pathology.

## Data Availability

All data analyzed in this article are publicly available in the published literature and are cited within our article and bibliography.

## Abbreviations

CP: cerebral palsy
MRI: Magnetic Resonance Imaging
SCPE: Surveillance of Cerebral Palsy in Europe
GMFCS: Gross Motor Function Classification System
MACS: Manual Ability Classification System
CFCS: Communication Function Classification System
EDACS: Eating and Drinking Ability Classification System
TD: typically developing
ROM: range of motion
MTU: muscle-tendon unit
PCSA: physiological cross-sectional area
ECM: extracellular matrix
IL-6: interleukin-6
TNF-α: tumor necrosis factor alpha
TGF-ß: transforming growth factor beta
SC: satellite cell
BoNT-A: botulinum neurotoxin-A
SDR: selective dorsal rhizotomy

## Footnotes

1 The fascicle length of a muscle is defined here as the length of the muscle in the direction of the muscle fibres or fascicles. We refrain from the term *fibre length* as fibres themselves terminate intrafascicularly; the term *fascicle length* better represents the functional length of a muscle measured from origin to insertion along the fibre direction.
